# *Trichuris suis* ova in inflammatory bowel disease-clinical challenges and translational pathways

**DOI:** 10.3389/fimmu.2026.1611690

**Published:** 2026-02-23

**Authors:** Su Zhou, Jiong Tang, Yu Wang

**Affiliations:** 1 Department of Pharmacy, Sichuan Gem Flower Hospital, Chengdu, Sichuan, China; 2 Department of Pharmacy, Chengdu Seventh People’s Hospital (Affiliated Cancer Hospital of Chengdu Medical College), Chengdu, Sichuan, China; 3 Department of Pharmacy, Mianyang Central Hospital, School of Medicine, University of Electronic Science and Technology of China, Mianyang, Sichuan, China

**Keywords:** challenges and prospects, inflammatory bowel disease, potential mechanisms, precision medicine, *Trichuris suis* ova

## Abstract

This article reviews the mechanisms and clinical trial findings of *Trichuris suis* ova (TSO) for inflammatory bowel disease (IBD).The results show TSO may exert potential effects on IBD via multiple pathways, yet no significant efficacy has been confirmed in clinical trials. Given its promising anti-inflammatory properties, further research is warranted. However, many knowledge gaps still exist in this field. Future trials should standardize study designs. Considering IBD complexity, priority should be given to precision medicine, with identifying TSO therapy’s target populations as a core step. Additionally, enhanced safety monitoring is essential to fully assess short- and long-term risks of TSO treatment. Given the inherent uncertainties of live biotherapeutics, multi-omics and gene-editing tools should be adopted to clarify TSO’s anti-inflammatory mechanisms and achieve its “artificial domestication”, enabling stable therapeutic performance across diverse clinical settings. The breakthroughs will deepen insights into IBD pathogenesis and advance microbiome-based interventions from empirical practice to the precision medicine era.

## 1 Introduction

With the socio-economic development of human society, the global incidence of Inflammatory Bowel Disease (IBD) continues to rise. IBD patients have multiple burdens: 10%-15% of IBD-related deaths are attributed to ulcerative colitis (UC)-associated cancer (1); patients’ quality of life is severely impaired, with symptoms such as fatigue, abdominal pain, and bloody stools being particularly prominent in females and Crohn’s disease (CD) populations, while gut microbial dysbiosis and immune imbalance exacerbate disease progression (2); pediatric patients may experience growth retardation; the global medical expenditure for IBD remains exorbitant, and recurrent flare-ups reduce labor productivity, constituting a heavy socio-economic burden (3).

Currently, chemical drugs and biologics are the most widely used agents for the IBD treatment, yet their limitations are evident. Immunosuppressants such as azathioprine and methotrexate can significantly increase risks of lymphoma, non-melanoma skin cancer, and urinary tract malignancies, while also causing bone marrow toxicity, hepatotoxicity, and infections (4, 5); glucocorticoids which used for induction remission, can cause osteoporosis, infection, and adrenal suppression with long-term use (6, 7); Biologics and novel small-molecule drugs may lead to severe infections or malignancies, while also increasing the risk of cardiovascular events and thrombosis (8, 9); 5-aminosalicylic acid (5-ASA) demonstrates limited therapeutic efficacy (6). In conclusion, current therapeutic approaches either have limited efficacy or may cause other more severe adverse consequences for patients. Therefore, exploring an efficient and low-toxicity therapeutic approach for IBD is an urgent priority in the field.

*Trichuris suis* (TS), which belong to the family *Trichuridae* and genus *Trichuris*, primarily parasitizes the cecum and colon of pigs (10). Its physiological metabolism adapts to the intestinal anaerobic environment, primarily relying on glycolysis to obtain energy (11). After pigs ingest *Trichuris Suis* Ova (TSO), larvae hatch in the intestine, penetrate the intestinal wall, migrate through tissues, and ultimately reside in the cecum and colon to mature into adults. Adults survive for months to years in pigs, during which females continuously release eggs that complete the life cycle through fecal excretion (12, 13). It is generally believed that as humans are not natural hosts for TS, its development in humans is restricted, resulting in shorter survival periods, which suggest favorable safety profiles (14, 15).

Infection with TSO is believed to modulate the host’s immune system (16, 17). It may treat immune-mediated diseases such as IBD through multiple mechanisms (18, 19), and this application initially derives from the “hygiene hypothesis”.

At present, TSO is the most well-studied helminth for IBD treatment. The essence of TSO therapy’s value lies in its potential to rectify immune dysregulation through mimicking natural symbiosis. It offer a physiologically aligned alternative that may circumvent the interpatient variability in drug efficacy and toxicities associated with conventional therapies. Theoretically, TSO therapy holds significant promise. Our research systematically analyzes the potential mechanisms, and reviews existing clinical trials of TSO in IBD treatment. It not only analyzes variations in results and core conclusions across trials, but also focuses on examining inherent limitations. Based on these and integrating clinical practical needs, it also clarifies current challenges in clinical application translation, while proposing targeted, feasible solutions. Ultimately, our work aims to facilitate the early, individualized, precision-based and standardized use of TSO in IBD clinical management.

## 2 Mechanistic insights and unresolved questions

IBD is a chronic inflammatory disease of the gastrointestinal tract. Its pathogenesis is believed to be linked to genetic, gut microbial, environmental, and immune factor (20, 21). Among these contributors, immune dysregulation is recognized as the most critical factor. Current evidence indicates that genetic, gut microbial, and environmental factors most likely induce IBD by disrupting immune homeostasis. Meanwhile, immune factors play a pivotal role in IBD treatment, as many currently available therapeutics exert their effects by targeting aberrant inflammatory signaling pathways.

UC and CD are widely accepted to be driven by exaggerated Th1(T helper type 1 cells)/Th17(T helper type 17 cells) immune responses (21). Nevertheless, subtle differences distinguish the two conditions. For example, Th9 cells(T helper type 9 cells) are primarily involved in the pathogenesis of UC (20). Additionally, there may be potential differences in the role of IL-17 between in UC and CD (22). Regulatory T cells (Treg cells) sustain immune homeostasis via multiple mechanisms, especially within the intestinal immune microenvironment (23). Beyond their immunomodulatory roles, Treg cells also exhibit non-immunomodulatory functions, such as mediating tissue repair through the secretion of the growth factor amphiregulin (24). The development of IBD is thought to be associated with functional impairments and numerical reductions in Treg cells (25, 26). Collectively, the onset of IBD is currently believed to be predominantly driven by exaggerated Th1/Th17 immune responses, underpinned by dysregulation between Th1/Th17 and Th2 (T helper type 2 cells) immune responses, and functional and numerical deficits in Treg cells.

Numerous parasites have been explored as potential agents for IBD. Specifically, several helminths, including *Trichuris, Trichinella, and Ancylostoma*, have been regarded as promising candidates. The effects of various helminths have been validated in animal experiments, such as *Schistosoma mansoni, Trichinella* sp*iralis, Trichuris muris, Trichuris trichiura*, and *Heligmosomoides polygyrus*. However, due to multiple factors, research on these organisms remains confined to the preclinical stage. To date, only two live helminths have progressed to human clinical trials, TS and *Necator americanus* (27, 28).

Similar to TS, the therapeutic efficacy of *Necator americanus* against IBD is likewise attributed to its immunomodulatory properties, as their common foundation is based on the “Hygiene Hypothesis” (27, 29). And they are most likely to regulate immune responses by stimulating the regulatory cytokine network primarily produced by Tregs (30), including modulating the balance between Th1/Th17 and Th2 immune responses. However, owing to the divergence in antigenic profiles and secreted products between them, whether their specific immunomodulatory mechanisms are consistent requires further experimental validation, as in the field, the mechanisms of effect by different helminths are rarely considered to be identical (31).

TS is the most commonly used, attributed to its demonstrated advantages over Necator americanus. First, TS’s anti-inflammatory and immunomodulatory efficacy in humans has been well validated. Besides IBD, it has also exhibited some effect for other autoimmune diseases. Its roles and mechanisms are also the most extensively studied. Then, unlike other parasites, humans are not regarded as the natural hosts of TS, rendering it unable to persist long-term in the human body. In contrast, *Necator americanus* requires only a single administration, as humans are its natural hosts. Chronic infection with it may induce intestinal bleeding, anemia, and nutrient deficiencies, which may be detrimental to the long-term health of patients (32, 33). Furthermore, this helminth can proliferate in human, a process largely influenced by factors such as the patient’s immune competence. It makes controlling helminth burden and invasiveness is more challenging. More importantly, TS is administered orally as TSO, which boasts a rapid action with generally mild and stable efficacy. In contrast, after *Necator americanus* inoculation, it takes approximately six weeks for the larvae to reach the intestinal tract, and longer period may be required to exert immunomodulatory effects. This indicates that patients with active disease, who demand prompt responses, are not ideal candidates for *Necator americanus*. Meanwhile, the early phase of *Necator americanus* infection (approximately 12 weeks post-inoculation) is characterized by a marked Th2 response. Initially, larval migration can induce serpiginous vesicular skin lesions accompanied by intense pruritus; subsequently, it may exacerbate rather than alleviate intestinal inflammation and trigger systemic acute inflammatory responses (28, 34).

**Figure 1:**
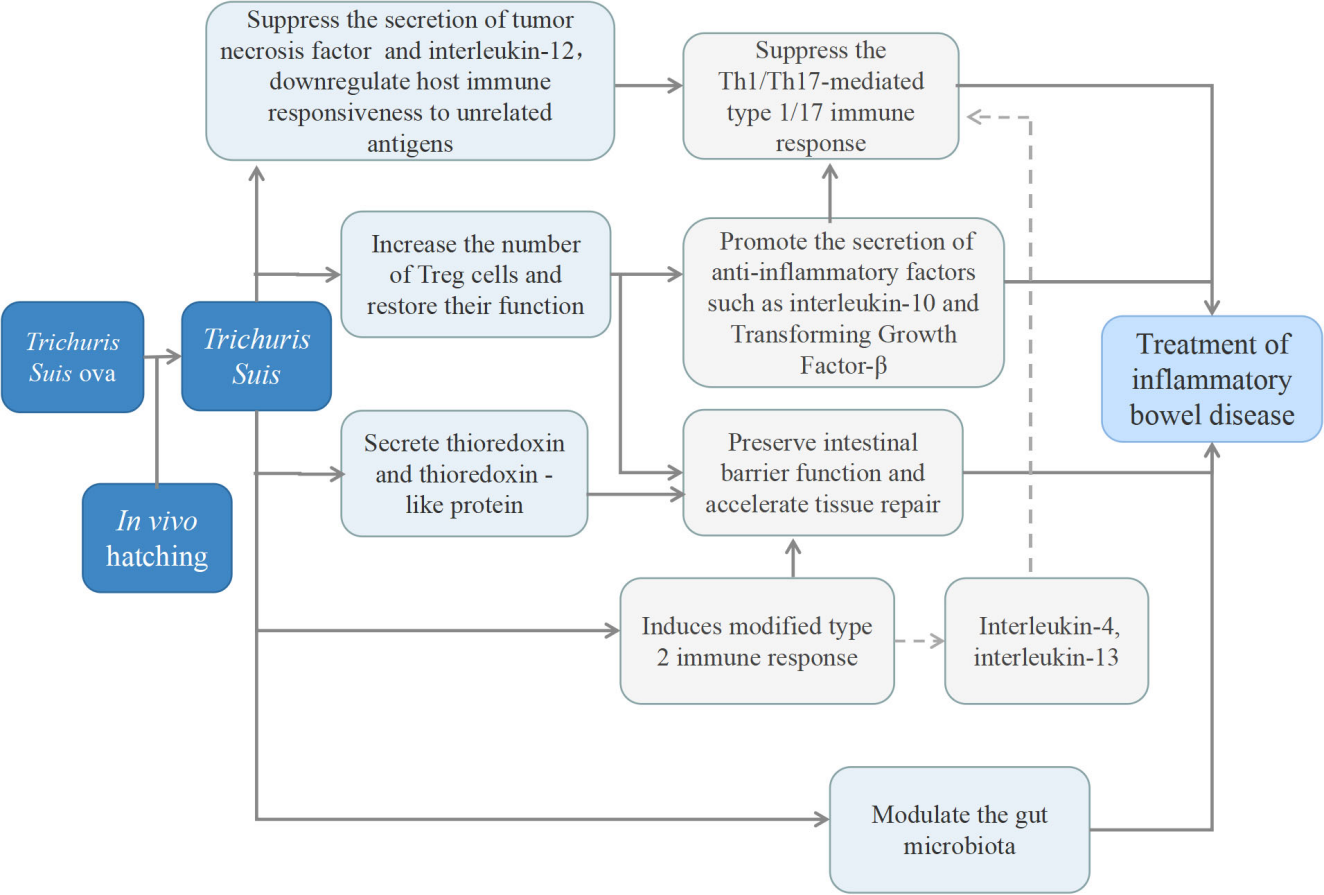
The main potential mechanism of *Trichuris suis* ova in the treatment of inflammatory bowel disease.

The specific mechanisms through which TS exerts effects on IBD remain inadequately explored. To date, mechanisms of TS-mediated IBD treatment are relatively scarce. Based on existing evidence, it is hypothesized to involve the synergistic crosstalk of multiple pathways (**Figure 1**). Potential mechanisms include: it can suppress the secretion of tumor necrosis factor (TNF) and interleukin-12 (IL-12) via the secretion of prostaglandin-E2 (PGE2) or other pathways, thereby promoting the polarization of dendritic cells toward a Th2 cell-inducing phenotype (35–37). Additionally, activating Rab7b protein to facilitate the degradation of Toll-like receptor 4 on the cell, which in turn mitigates the conversion of CD4^+^T cells to Th1/Th17 cell subsets induced by endotoxins derived from the intestinal flora. Meanwhile, TS can downregulate host immune responsiveness to other unrelated antigens (38), and also can suppresses the expression of proinflammatory genes in the intestinal mucosa while stimulating the production of Th2 response, inhibiting Th1/Th17 response (16, 39). Furthermore, TS can induce the generation of regulatory T cells (Tregs) and promote the secretion of immunomodulatory molecules (transforming growth factor-β [TGF-β], interleukin-10 [IL-10]), which contributes to maintaining host mucosal immune homeostasis (16, 40). Apart from these, a serine protease secreted by TS can regulate mast cell-related protease-mediated host immune responses in the intestinal mucosa (41). It also secretes thioredoxin and thioredoxin-like protein which exhibit antioxidant activity and facilitate tissue repair (42). Notably, TS may also modulate the gut microbiota, although the underlying mechanisms remain poorly elucidated (43), and this interaction maybe bidirectional. For example, helminths may induce alterations in the abundance and diversity of the gut microbiota by releasing antimicrobial peptides or modifying the nutritional microenvironment of local tissues. Conversely, the gut microbiota may promote helminth egg hatching and even regulate the immune system’s ability to clear parasites (44). *In vitro* studies, the excretory-secretory products (ESP) of adult TS have been shown to exhibit antibacterial activity, with Gram-negative bacteria (e.g., *Campylobacter* spp.*, Escherichia coli*) and Gram-positive bacteria (*Staphylococcus aureus*) showing sensitivity to ESP (45). It is critical to emphasize, however, that this conclusion is solely derived from *in vitro* experiments.

Furthermore, infection with various helminths, including TS, may induce changes in intestinal mucosal barrier function. Theoretically, Th2 cells exhibit significantly increased numbers and enhanced functional activity in this context, and their cytokines (particularly IL-13) together with IL-22 exert profound effects on colonic epithelial cell function. These effects include stimulating the differentiation of goblet cells and Paneth cells via associated mucus production and antimicrobial peptide expression, as well as activating anti-apoptotic signaling pathways. Additionally, accessory cells recruited and activated by Th2 response, especially alternatively activated macrophages, can promote mucosal healing (46). Meanwhile, TS can also contribute to the repair of damaged mucosa through the modulation of Treg cell activity (47, 48). However, empirical evidence has demonstrated that helminths, including TS, do not consistently exert a mucosal protective effect; instead, they may occasionally induce mucosal impairment (49, 50). *Trichuris muris*, a helminth species closely related to TS, secretes serine proteases that alter mucus barrier properties by degrading mucin components within the mucus gel, rendering it more porous (51). Similarly, TS encodes a repertoire of serine proteases (52), but whether they would show analogous effects remains to be elucidated. Thus, the impact of helminths on the intestinal mucosa is likely contingent upon factors such as species, distinct survival states, environmental conditions, and host-specific traits, etc.

Notably, despite the multiplicity of proposed mechanisms, none adequately account for the lack of efficacy of TS in certain clinical trials. Therefore, further research is required to identify the factors that modulate the functionality of these mechanisms, representing a critical knowledge gap that warrants further exploration.

Currently, in addition to its use in the treatment of IBD, TSO has also shown pharmacological effects in multiple sclerosis (MS),allergic rhinitis (AR) and autism spectrum disorder (ASD). For multiple sclerosis, TSO treatment has been reported to reduce pro-inflammatory factors, elevate anti-inflammatory factor concentrations, lower disease severity scores, and improve neurological imaging outcomes (53–56); For allergic rhinitis, it has been shown to reduce the number of days during the grass pollen season when participants required rescue medication (57); Although these therapeutic effects are generally mild to moderate. In adult autism spectrum disorder, TSO has exhibited large effect sizes specifically in improving rigidity and repetitive behaviors (58). Notably, all these positive outcomes are attributed to TSO’s inherent anti-inflammatory and immunomodulatory activities.

Compared with TS-mediated mechanisms in treating other diseases, the actions in IBD may exhibit distinct nuances. MS is also characterized by dysregulation between Th1/Th17 and Th2 immune responses, alongside functional and numerical deficiencies in regulatory Treg cells (59–61). Thus, the mechanism of TS in MS is generally considered analogous to that in IBD. The key distinction lies in the anatomical locus of inflammation: MS is centered on central nervous system inflammation, whereas IBD is driven by Th1/Th17-dominant inflammation and intestinal mucosal barrier impairment. This underscores the need to clarify whether TS exerts local or systemic effects-particularly whether the effect and ESP can cross the blood-brain barrier (37, 60). Notably, current clinical trials suggest such concerns may be unwarranted, as TSO has demonstrated partial efficacy in MS. (55, 56). ASD is a heterogeneous group of multifactorial neurodevelopmental conditions. Its pathogenesis involves multiple contributors, with the immune hypothesis emerging as a key mechanism. Current evidence indicates that T lymphocyte dysregulation in ASD encompasses an abnormal T helper-suppressor cell ratio, systemic Treg cell depletion, and aberrant cytokine release (62, 63). For example, increased hyperactivity correlates with low levels of anti-inflammatory cytokines (IL-10, TGF-β) and elevated proinflammatory cytokines (IL-1β, IL-6) (64, 65), while a Th1 response is associated with more severe developmental impairments (66). Additionally, ASD has been linked to intestinal barrier dysfunction and gut microbiota dysbiosis (67). Thus, analogous to IBD, TS is hypothesized to treat ASD through a synergistic mechanism involving Th2-response induction, inhibition of Toll-like receptor-mediated Th1/Th17 activation, intestinal barrier enhancement, and gut microbiota modulation (17, 58). However, similar to MS, TS colonizes the intestinal tract, and the specific pathway by which it modulates gut-brain axis function remains elusive.

In allergic diseases, *TS* may exert distinct mechanisms. Excessive Th 2 responses—hallmarks of asthma and allergies-represent an overactivated state that typically does not involve Th1/Th17 activation (17, 68). In this context, both Th1 responses and Treg cell function are markedly suppressed. Specifically, insufficient Treg-mediated control drives Th2 overactivation. Unlike classical type 2 immunity, helminth infection induces a “modified type 2 immune response” that fails to trigger allergic inflammation following allergen exposure (69). This modified response is attributed to helminth-induced enhancement of Treg and regulatory B cell (Breg) activity, coupled with upregulation of the key anti-inflammatory cytokines IL-10 and TGF-β (68). Treg activation augments anti-inflammatory cytokine release, while Breg activation inhibits pathogenic antibody production (e.g., IgE in allergies), elevates allergen-specific IgG4 levels, and attenuates autoreactive B cell activation (69–71).

Collectively, helminths including TS exhibit disease-specific mechanisms of action, reflecting their capacity to comprehensively restore immune homeostasis by regulating Th1/Th17-Th2 balance, while concurrently enhancing Treg cell number and function.

**Figure 2:**
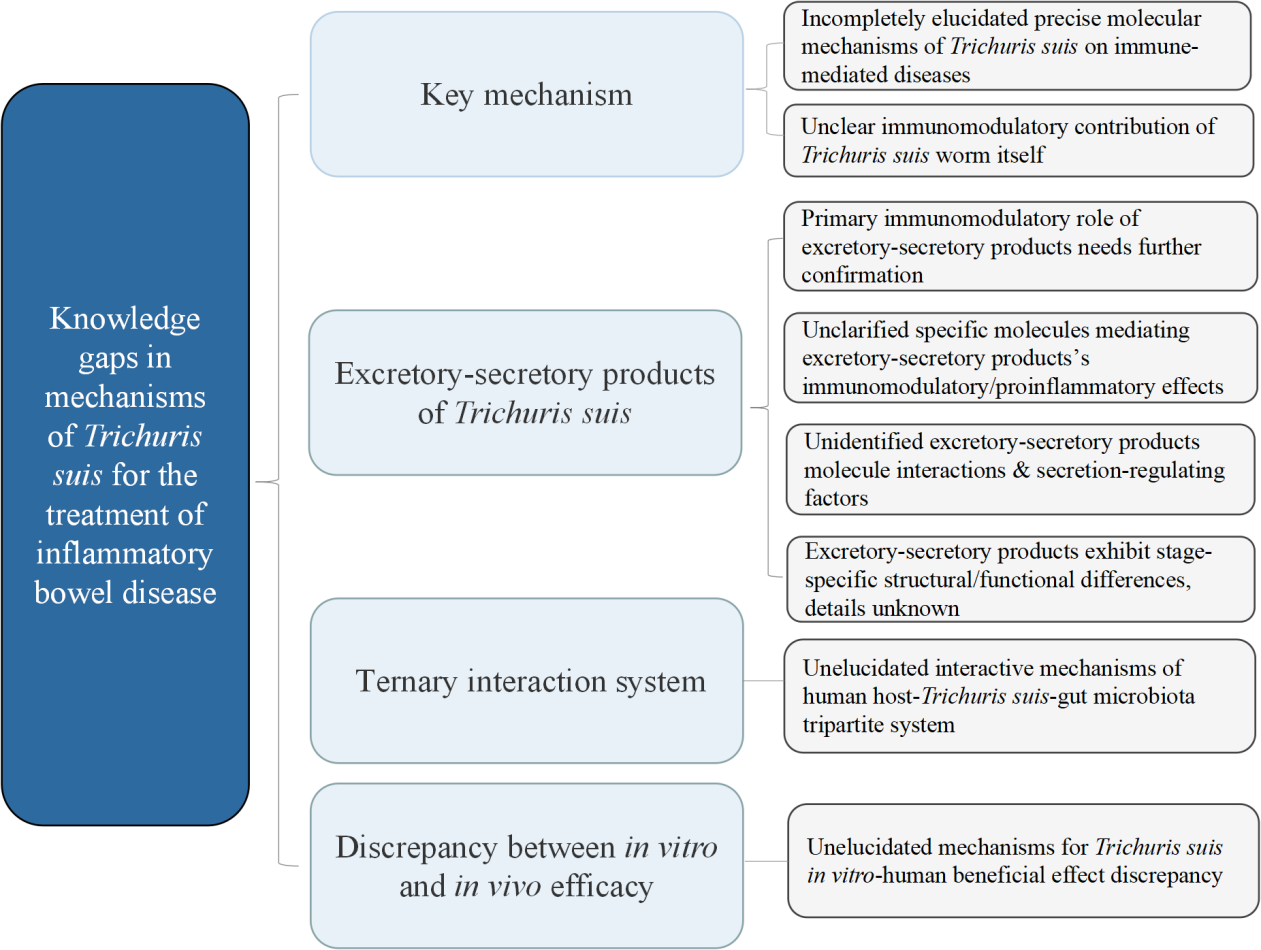
Knowledge gaps in mechanistic research on *Trichuris suis* ova for inflammatory bowel disease therapy.

Nevertheless, critical knowledge gaps persist. First, the precise mechanisms underlying TS’s effects on immune-mediated diseases remain incompletely elucidated. While ESP are implicated as primary immunomodulatory mediators, the potential contribution of the TS worm itself remains unclear. Regarding ESP, the specific molecules responsible for immunomodulation versus proinflammatory effects, their interactions, and the contextual factors governing ESP secretion profiles require clarification. Additionally, alternative mechanisms (e.g., TS-gut microbiota crosstalk in humans) have not been fully explored. Furthermore, TS ESP functions vary across life cycle stages (72), but the molecular basis for these stage-specific differences is unknown. Most importantly, whether TS consistently exhibits beneficial effects in humans—mirroring its *in vitro* activity—requires further validation (**Figure 2**).

## 3 Effectiveness and safety profiles of TSO in IBD treatment

### 3.1 Ulcerative colitis

Overall, the therapeutic role of TSO in UC remains poorly defined, as controlled trials—even those with rigorous design—have failed to demonstrate efficacy in primary endpoints. In a single-arm trial, TSO combined with other medications induced remission in most UC patients (73). Although the results appear promising, the small sample size and absence of a control group limit their interpretability. The controlled trials have yielded primary endpoints that are less favorable than anticipated. Summers assessed UC patients with prolonged disease duration and reported improvements in UCDAI scores and clinical symptoms. However, the primary outcome of induction remission showed no significant difference (74). Another trial (NCT01953354) enrolled UC patients with left-sided colitis but had imbalances in patients’ baseline characteristics —most notably in the use of concomitant medications. At the 12-week follow-up, no significant differences were observed between the groups in the rates of remission, response, and mucosal healing (75). In a recent randomized controlled trial comparing TSO with placebo, no significant between-group differences were found in remission or response rates at 24 weeks. In a subgroup of patients who did not receive corticosteroids during the trial, transient symptomatic remission was observed at week 12, and partial improvements in Mayo scores were noted at weeks 14 and 18. Nonetheless, these isolated findings lacked logical consistency and may may be indicative of statistical bias (76) (**Table 1**). Common limitations across these trials include small sample sizes, suboptimal randomization procedures, inadequate blinding, imbalances in baseline characteristics, and concomitant use of other therapies (e.g., mesalamine, corticosteroids, azathioprine). Consequently, even positive results can only position TSO as an adjunct to conventional therapies rather than a standalone induction agent. Moreover, heterogeneity in outcome measures-such as the UCDAI versus Mayo scores- complicates cross-study comparisons.

**Table 1:** Clinical trials characteristics of *Trichuris suis* ova for Inflammatory bowel disease.

Researchers and reference	Disease	Population	Country/region	Study design	Dosage of TSO	Outcome measures	Main conclusions	Limitations
Prosberg MV, 2024 (76)	UC	119 patients with moderately active UC (Mayo score 6–10)	Denmark	Single-center, randomized, double-blind, placebo-controlled, phase 2b clinical trial	7500 TSO every 2 weeks for 24 weeks	Primary outcome: Clinical remission (Mayo score ≤2) at 24 weeks; Secondary outcomes: Clinical response, corticosteroid-free remission, endoscopic remission, etc.	TSO was not superior to placebo in achieving clinical remission at 24 weeks, but temporary symptomatic remission was observed at 12 weeks in patients not requiring corticosteroids.	Concomitant medication confounding; Unconsidered dietary, environmental and psychological factors;High dropout rate
Summers RW, 2005 (16)	CD	29 patients with active CD (CDAI ≥ 220)	United States	Open-label study	2500 TSO every 3 weeks for 24 weeks	CDAI score, clinical remission (CDAI < 150)	79.3% of patients responded, and 72.4% achieved remission, with no adverse events.	Uncontrolled trial;Concomitant medication confounding;Unconsidered dietary, environmental and psychological factors;Incomplete outcome measure assessment;Small sample size;Complete lack of blinding
Schölmerich J, 2017 (77)	CD	252 patients with mildly to moderately active ileocolonic CD	Europe	Multicenter, randomized, double-blind, placebo-controlled trial	250, 2500, 7500 TSO every 2 weeks for 6 doses	Clinical remission (CDAI < 150) at 12 weeks, clinical response, changes in inflammatory markers	No TSO dose showed superiority over placebo, but TSO was safe and well-tolerated.	Concomitant medication confounding; Unconsidered dietary, environmental and psychological factors;High dropout rate;Incomplete outcome measure assessment,Short observation period
Jaeger SU, 2018 (78)	CD	207 participants from a previous clinical trial (Schölmerich et al., 2017)	Germany	Retrospective genotyping study based on a randomized, double-blind, placebo-controlled clinical trial;	250, 2500, 7500 TSO every 2 weeks for 6 doses	Clinical remission (CDAI < 150) at 12 weeks	No consistent significant association was observed between the 6 NOD2 variants (or their haplotypes) and clinical outcomes (remission/response) of TSO treatment	Concomitant medication confounding; Unconsidered dietary, environmental and psychological factors;High dropout rate;Incomplete outcome measure assessment,Short observation period;The randomization non-stratified by genotype
Sandborn WJ, 2013 (79)	CD	36 CD patients (divided into 3 groups: 500, 2500, 7500 TSO vs. placebo)	United States	Randomized, double-blind, placebo-controlled, dose-escalation trial	Single dose of 500, 2500, or 7500 TSO	Incidence of adverse events, changes in gastrointestinal symptoms	Single-dose TSO (up to 7500 ova) was safe, with no dose-dependent adverse reactions.	Concomitant medication confounding; Unconsidered dietary, environmental and psychological factors;Incomplete outcome measure assessment;Small sample size
Summers RW, 2003 (73)	IBD	4 CD patients and 3 UC patients	United States	Open-label study	2500 TSO (single dose, with maintenance treatment in some patients)	CDAI, SCCAI, IBDQ score	All patients showed clinical improvement; single-dose effect was temporary, while maintenance treatment prolonged remission.	Uncontrolled trial;Concomitant medication confounding;Unconsidered dietary, environmental and psychological factors;Incomplete outcome measure assessment;Small sample size;Complete lack of blinding
Summers RW, 2005 (74)	UC	54 patients with active UC (UCDAI ≥ 4)	United States	Randomized, double-blind, placebo-controlled trial	2500 TSO every 2 weeks for 12 weeks	Clinical improvement (UCDAI reduction ≥ 4) at 12 weeks	The improvement rate was 43.3% in the TSO group vs. 16.7% in the placebo group (P = 0.04), and TSO was safe.	Short observation period;Concomitant medication confounding;Unconsidered dietary, environmental and psychological factors;Incomplete outcome measure assessment;Small sample size
Stephen H, 2012 (75)	CD	250 patients with moderately to severely active CD	United States	Randomized, double-blind, placebo-controlled, multicenter trial	7500 TSO every 2 weeks for 10 weeks of 6 total doses	Reduction in CDAI by ≥ 100 points	The 12-week response rates showed no significant difference between the TSO group (46.4%) and the placebo group(44.0%)	Short observation period;Concomitant medication confounding;Unconsidered dietary, environmental and psychological factors,Incomplete outcome measure assessment
Silver N, 2013 (80)	UC	Planned enrollment of 16 patients with left-sided colitis (trial terminated)	United States	Randomized, double-blind, placebo-controlled trial	7500 TSO every 2 weeks for 10 weeks for a total of 6 total doses	Clinical response (Mayo score reduction ≥ 3 points and ≥ 30%) and remission rate at 12 weeks	At the 12-week follow-up, no significant differences were observed between the groups in the rates of remission , response, and mucosal healing.	Short observation period;Concomitant medication confounding;Unconsidered dietary, environmental and psychological factors

TSO, *Trichuris suis* ova; UC, Ulcerative Colitis; CD, Crohn’s Disease; IBD, Inflammatory Bowel Disease; NOD2, Nucleotide-binding oligomerization domain 2.

### 3.2 Crohn’s disease

Consistent with observations in UC, therapeutic strategies in CD exhibit analogous roles. An early single-arm trial indicated potential therapeutic efficacy. In a study by Summers (73), 3 of 4 CD patients achieved remission. In a subsequent another study, TSO associated with improved response rates (CDAI reduction >100 or CDAI <150) and remission rates (CDAI <150) at week 24.Responders exhibited a mean CDAI decline of 177.1 points (16). The intervention appears to be effective; however, it lacked a control group.

Controlled trials have failed to demonstrate efficacy. In another trial (NCT01576471) involving 250 patients with moderate-to-severe CD, the 12-week response rates showed no significant difference between the TSO group (46.4%) and the placebo group(44.0%) (80). Another trial enrolling 252 patients with mild-to-moderate CD tested escalating TSO doses (250, 2500, or 7500 eggs administered every two weeks for 12 weeks). All TSO dose groups showed no superiority over the placebo for the primary outcome. Furthermore, no dose-response relationship was observed across the tested dose range (77). Like the trials in ulcerative colitis, those in CD were further hampered by methodological flaws, such as inadequate statistical power, suboptimal randomization and blinding, and confounding effects from concomitant medications.

In both UC and CD trials, TSO has demonstrated a good safety profile, with no serious adverse events reported other than transient gastrointestinal symptoms, even in a dedicated safety study (79) (**Table 1**).

## 4 Current clinical evidence limitations and gaps

The current clinical evidence does not strongly support TSO as an effective therapy for IBD. However, its promising preclinical data (including animal studies), potential benefits observed in secondary endpoints, and the limitations of existing pharmacotherapies (e.g., toxicity, variable efficacy) continue to maintain interest in TSO treatment. Meanwhile, as previously noted, TSO has demonstrated certain therapeutic efficacy in other human autoimmune diseases. Given these considerations, TSO may remain a promising therapeutic strategy for IBD. Negative trial results may stem from methodological limitations (e.g., heterogeneous populations, confounding concomitant therapies) rather than inherent inefficacy. Future trials should address these flaws through more rigorous design. Thus, we will discuss the limitations in existing clinical trials, technical bottlenecks, and potential approaches to overcome the dilemma.

### 4.1 Limitations of existing clinical trials

#### 4.1.1 Uncontrolled studies

Currently, uncontrolled studies are mainly focused on preliminarily exploring the efficacy of TSO in treating IBD. Moreover, more emphasis is placed on observing its tolerability and safety. The efficacy of TSO appears promising. Notably, unlike many other diseases, both UC and CD may exhibit spontaneous symptomatic remission–attributable to their natural disease course or placebo effects–as demonstrated in other trials (81). While such remissions often occur at the symptomatic level, they do not necessarily reflect endoscopic or histological improvement, and their overall rates remain limited. UC may exhibit a spontaneous remission rate of 55%–65% in the first year, which can persist over time (82); in CD, the remission rate is lower, reaching up to 50% (83). During longer observation periods, patients with UC and CD frequently experience recurrent acute flares and episodes of spontaneous remission, reflecting the inherent chronic and relapsing nature of IBD. Therefore, uncontrolled studies alone provide insufficient evidence to interpret efficacy outcomes. Research evaluating therapeutic efficacy should avoid relying solely on uncontrolled trial designs.

#### 4.1.2 Sample size

Existing clinical trials, whether uncontrolled or controlled, have sample sizes ranging from a few cases to over 100 participants per study arm. The modest therapeutic effects of TSO in current studies indicate that the existing sample sizes are insufficient to draw definitive conclusions. Small sample sizes are typically associated with inadequate statistical power and challenges in detecting significant differences. Additionally, considering the complexity of IBD—which encompasses patient baseline characteristics such as genetic susceptibility (84–86), clinical phenotypes, disease severity, and factors influencing treatment response (87)—small sample sizes may inadequately represent the heterogeneous patient population. Furthermore, small-scale studies pose challenges to subgroup analyses, make it difficult to identify potential beneficiary subgroups, and restrict the exploration of the interaction network among the host, parasite, and microbiota. Therefore, large-scale multicenter clinical trials are essential.

#### 4.1.3 Additional determinants

Almost all current trials have failed to incorporate socio-environmental, sanitary and dietary factors into their designs. This limitation is significant because environmental conditions, sanitation levels, and socioeconomic status are well-recognized contributors to IBD pathogenesis or disease reactivation (88). Neglecting to account for these factors during trials may lead to confounding results. It is worthing noting that post-diagnosis dietary habits significantly impact the short-term prognosis in IBD patients. Certain dietary regimens, such as the classic or modified Carbohydrate Diet, the Mediterranean Diet, or the low-FODMAP Diet, have shown potential in inducing or maintaining remission (89–93).

Psychological factors also deserve attention since they may have a wide-ranging impact on intestinal function, mucosal barrier repair, gut microbiota composition, as well as both humoral and cellular immune responses (94–96). Clinical evidence suggests that stress, anxiety, and depression may precipitate IBD relapse or disease deterioration, whereas mitigation of these psychological factors could facilitate symptom remission (97, 98). Although existing trials have attempted to balance or minimize confounding variables (e.g., ethnicity, age of onset, gender, smoking status, disease duration, affected anatomical regions, and concomitant medications), it is necessary that future trial designs systematically integrate socio-environmental, sanitary, dietary patterns, and psychological factors as covariates.

#### 4.1.4 Outcome measures

In the aforementioned clinical trials, disparities in study durations and variations in disease assessment scales have led to heterogeneous criteria for outcome evaluation. For example, different scoring systems, including the Inflammatory Bowel Disease Quality of Life Questionnaire (IBDQ), Mayo Clinic Score, and Ulcerative Colitis Disease Activity Index (UCDAI), are used to assess disease severity and therapeutic efficacy in UC. Notably, these scoring systems also differ in their focus: the IBDQ and UCDAI are not currently recommended as primary outcome measures for UC. Pooled analysis of such divergent metrics may introduce bias (13). Furthermore, inconsistencies in study durations pose challenges, as short-term studies, by their nature, lack the robustness of long-term investigations in evaluating overall therapeutic efficacy and safety. However, with the growing understanding of IBD and increasing standardization of evaluation criteria, future trials are expected to demonstrate substantial advancements in this field. Of particular note, mucosal healing—a recognized long-term treatment target in IBD (99)—is frequently overlooked, especially in CD research. Although current methods for assessing mucosal healing are limited by subjective interpretation bias, insufficient consideration of disease extent, and suboptimal composite scoring systems (100), the omission of this outcome may significantly undermine the credibility of study results. While standardization of IBD management aims to address this gap, whether mucosal healing achieved through microbial therapies is clinically equivalent to that achieved via conventional pharmacological approaches remains unclear. Currently, no evidence supports comparable clinical long-term outcomes between these treatment modalities. Meanwhile, as live organisms, microbial therapies may induce variable immunomodulatory responses—potentially fluctuating between immune regulation and activation—rather than sustained, consistent modulation.

Another emerging concern is the detection of histologic inflammatory activity in cases with endoscopically confirmed mucosal healing (100). This low-grade inflammation highlights the potential value of histologic assessment for monitoring disease progression: while it demands greater technical and resource input, it can reduce the subjective bias inherent in macroscopic evaluations. Although not yet formally endorsed as an IBD outcome measure (99), histologic remission is gaining prognostic importance due to its direct correlation with mucosal or submucosal inflammation. It may better predict relapse rates and long-term outcomes (particularly in UC, though evidence in CD remains inconclusive) and could be considered a target for achieving deep remission (101). Current perspectives propose histologic healing for UC and transmural healing for CD as distinct endpoints (102), reflecting a paradigm shift toward sustained control of chronic inflammation to improve long-term outcomes, such as reduced surgical risk (100). Future studies evaluating TSO should incorporate histologic remission assessments. However, theoretically, parasite-based long-term therapies—as non-native microbiota—may sustain low-grade mucosal inflammation rather than induce complete histologic remission, given their higher antigenic burden compared to commensal bacteria. If microbial therapies fail to achieve full histologic remission, whether their clinical outcomes are comparable to those of conventional drugs remains uncertain and requires rigorous investigation.

#### 4.1.5 Concomitant therapy

In current studies, only a very small proportion of patients received TSO as monotherapy. Among concomitant therapies, 5-aminosalicylic acid (5-ASA) agents were the most frequently administered, followed by glucocorticoids. Thus, existing evidence does not support using TSO as a standalone treatment for IBD; rather, its efficacy likely depends on combination with other agents tailored to disease severity. Even in studies reporting positive outcomes, these results may only reflect a potential synergistic role of TSO in IBD management. Notably, 5-ASA monotherapy has been shown ineffective in inducing or maintaining remission in CD (103, 104); nevertheless, many trial participants still received this agent. It is evident that the effects of concomitant therapies on TSO’s efficacy not be ignored before interpreting study results. To date, no studies have investigated potential interactions between 5-ASA and TS. Theoretically, sulfasalazine —a 5-ASA formulation— contains a sulfonamide component that may disrupt folate metabolism in helminths without directly killing them, thereby altering TS growth and metabolic behavior. Similar effects have been observed in nematode models (105). Further studies are necessary to evaluate this interactive mechanism.

Additionally, animal studies indicate that glucocorticoids and immunosuppressants may significantly antagonize the immunomodulatory effects of TS by disrupting its survival kinetics and altering host immune microenvironment (106). With the rising utilization of biologic agents in IBD management, their interactions with TS remain uninvestigated. Therefore, future clinical trials should first define their primary objective: to evaluate the standalone therapeutic potential of TSO in IBD or its utility as an adjunctive therapy. Trial designs should be meticulously structured to align with the specified the research aims.

#### 4.1.6 Safety profiles

While TS has been considered safe in clinical trials due to its assumed inability to complete its life cycle in humans and low invasiveness, emerging evidence challenges this assumption. A study indicates that TS can colonize the human colon, developing into adult worms similar to those in its natural porcine host, with unembryonated eggs detected in human feces (39). Furthermore, confirmed cases of TS infection in humans have been reported when using TSO (107). Although these observations partially fulfill Koch’s postulates, together they suggest that TS may exhibit greater infectivity than previously recognized (108). Notably, the infected patients had received high-dose corticosteroids, immunosuppressants, or infliximab prior to TS administration, highlighting the need for caution regarding infection or larval migration risks in immunocompromised populations. The mechanism may be that immunosuppression impairs Th2 responses, thereby compromising the effective clearance of larvae; the damaged mucosal barrier function facilitates the invasion of larvae into deep tissues; the reduced immune surveillance capacity may permit the larvae to complete their developmental cycle (109). Meanwhile, the damage caused by worm eggs traversing the gastrointestinal epithelium can result in systemic translocation of bacteria which also merits attention (110, 111). As in these patients, the systemic infection may be more hazardous than the helminth infection itself. Thus, TSO therapy is not recommended for immunocompromised individuals (including those with HIV, malignant tumors, drug-induced immunodeficiency, etc.). If administration is deemed absolutely necessary, close monitoring should be implemented for signs and symptoms associated with TS infection, and anthelmintic treatment should be promptly initiated when clinically indicated. Meanwhile, regular monitoring should also be conducted to ensure the absence of TS colonization and infection.

Additionally, TSO does not consistently exhibit therapeutic effects over potential harmful actions. Animal trials reveal that baseline immune status critically influences outcomes in IBD models. Immunosuppressed rabbits developed exacerbated intestinal inflammation following TSO exposure (106), though whether this is caused by invasive infection or hypersensitivity reactions remains unclear (112). From a mechanistic standpoint, invasive infection appears more plausible based on the population-level observations, as hypersensitivity reactions typically manifest sporadically. These safety concerns have been formally recognized in regulatory documents of the European Medicines Agency (113).

Due to insufficient data, it remains unclear whether TS harbors carcinogenic potential analogous to that exhibited by other parasites. But like bacteria and viruses, helminths are increasingly recognized as being associated with cancer (114–117). The potential mechanisms include: indirect modulation of host immune responses; direct carcinogenicity; chronic mechanical tissue damage and subsequent repair; perturbations in microbiota homeostasis; and antigen cross-reactivity. *Trichuris.* spp are generally regarded as having minimal carcinogenic potential. However, theoretically, even residual *Trichuris* components (e.g., dead *Trichuris* bodies and eggs) within the host could elicit local foreign body reactions or sustained inflammation. This damage-repair cycle promotes intestinal tumor initiation, albeit considerably less potently than those induced by trematodes. Furthermore, as previously noted, *Trichuris* does not uniformly exert anti-inflammatory and immunomodulatory effects in all individuals. Sometimes, it may instead elicit chronic inflammation and sustained tissue injury. Thus, *Trichuris* may theoretically contribute to intestinal tumorigenesis which is supported by some evidence. *Trichuris muris* has been demonstrated to promote colonic hyperplasia, the formation of aberrant crypts foci and adenoma precursors, and inflammatory cell infiltration within the colonic lamina propria in mice. Moreover, it can induce neoplastic alterations analogous to those seen in mice exposed to the carcinogen azoxymethane (118, 119). Limited evidence indicates that *Trichuris trichiura* may also be linked to specific intestinal malignancies (120), moreover, theoretically, this risk indeed exists. During colitis, the T cells producing only interleukin-17 were abundant, whereas after *Trichuris* infection more multifunctional T cells are induced, secreting cytokines including interleukin-22 (121). The accumulation of interleukin-17 and interleukin-22 coexpressing cells is associated with colorectal cancer (122). While the immune response elicited by TS in humans is typically weaker than that in its natural porcine host, given the close phylogenetic relationship among *Trichuris trichiura*, *Trichuris muris*, and TS (123, 124), the potential for cancer development mediated by its induced chronic inflammation cannot be entirely discounted. Notably, unlike healthy individuals, patients with IBD may be receiving concurrent immunosuppressive therapy. This may compromise the immune system’s ability to monitor and control TS, potentially enabling this parasite to deviate from its normal life cycle and result in unanticipated consequences.

Furthermore, some helminths do not induce cancer at their site of colonization but instead at distant anatomical sites (125). The mechanism remains poorly understood. Whether TS exhibits similar properties is currently unknown.

In summary, these risks should not be overlooked—particularly for individuals with preexisting intestinal mucosal damage or immunosuppression. Moreover, the long-term effects of TSO in humans remain largely unknown. For instance, gaps exist in understanding its complete *in vivo* lifecycle, migration patterns, and whether worm biomass or eggs persist in the intestinal wall to induce chronic inflammation. Based on current evidence, IBD patients with compromised immunity or suboptimal baseline health may not be suitable candidates for TSO*-*based therapy. Meanwhile, as a biological therapeutic agent, the carcinogenic and mutagenic potential of TSO warrants rigorous evaluation, particularly in individuals receiving long-term treatment or with immunodeficiency. This necessitates incorporating additional clinical follow-up data and toxicological investigations to furnish critical evidence for the risk-benefit balance in its clinical application.

### 4.2 Technical bottlenecks

Contemporary clinical research on live biotherapeutic interventions encounters methodological limitations, as randomized controlled trials (RCTs) demonstrating limited capacity to establish causal relationships due to the inherent complexity of heterogeneous microbial interactions. Taking probiotics research as an example: even in well-designed RCTs for the same indication, conflicting results often emerge, including both positive and negative outcomes or seemingly contradictory positive results (126–129). The primary issue stems from treating live organisms as equivalents to conventional chemical drugs or biological agents, rather than recognizing them as dynamic living entities. This oversight disregards the diverse survival pressures exerted by factors such as manufacturing processes and fluctuations in host physiological conditions on live organisms, leading to context-dependent variability in their effects. Essentially, it overlooks the “behavioral plasticity” of these live organisms. Even with modern *in vitro* techniques, ensuring consistency in microbial products remains challenging, as there are still many production factors that are currently unknown or uncontrollable. (130, 131). Once administered *in vivo*, live organisms products encounter complex interactions with host factors— including gut microbiota composition, gastrointestinal motility, immune status, diet, genetics, environment, and psychological state. The human microbiome comprises over 1,000 microbial species, with each individual colonized by 10-20% of these taxa (132, 133)]. This personalized microbiota may impose differential survival pressures on exogenous microbes, leading to variable outcomes that could be beneficial, harmful, or even fluctuating (134, 135). Moreover, the interaction between the immune system and exogenous organisms-especially probiotics-remains poorly understood due to technical limitations in longitudinal, dynamic studies (132, 136). As a result, some skeptics argue that the therapeutic effects of probiotics are currently “unknown” (137).

Helminths, such as TS, exhibit greater complexities compared to single-celled probiotics. Their multicellular structure grants enhanced adaptability to adverse conditions and produces more diverse metabolites and secretory products (124, 138). However, this complexity demands advanced manufacturing and quality control protocols—processes that remain underdeveloped due to unresolved challenges in simpler probiotic formulations (139). Understanding TS-host interactions is particularly challenging, as it discussed in the section on mechanisms of TS for IBD treatment:TS secretes beneficial metabolites that directly modulate host cells (37), yet may also generate uncharacterized harmful substances when stressed. It can also interact bidirectionally with the gut microbiota, influencing microbial composition and potentially triggering antimicrobial responses (45, 140–143). Moreover, the mechanisms underlying TS-mediated immune regulation likely involve the cumulative effects of multiple pathways rather than a single factor (19, 52). The interconnected nature of these mechanisms may give rise to non-additive effects, substantially complicating the elucidation of causal relationships.

## 5 Promising clinical translation strategies​

Advancing TSO-based clinical research for IBD treatment requires fundamental investigations into its mechanisms. At present, although some progress has been made in mechanistic studies of TSO for IBD treating, these studies have not elucidate the mechanism comprehensively and macroscopically (144–147). They always only focus on one or a few targets. This may be one of the significant reasons why the effect is remarkable in basic research but the opposite occurs in clinical trials. Therefore, interactions of mechanisms should be carefully investigated. Meantime, given the multitude of influencing factors, *in vitro* studies should more accurately simulate the parasitic growth environment within the host, particularly considering individual immune profiles and gut microbiota diversity. In animal experiments, researchers must carefully address interspecies differences, as disparities in immune profiles and baseline gut microbiota between species are substantial and exert a significant impact on study outcomes. For outcome assessment, in addition to clinical symptoms, biochemical markers, and endoscopic remission, histological remission should also be included. Moreover, rigorous validation is required to determine whether TSO-induced endoscopic and histological remission are comparable in long-term clinical significance to those achieved by conventional therapeutics. Crucially, the survival rate of TSO must be included as a key endpoint, as live TSO—similar to probiotics—likely yields stronger therapeutic effects than dead organisms, despite the latter’s residual biological activity. To clarify how multifactorial variability influences therapeutic outcomes, TSO-IBD research should employ multi-omics technologies (e.g., transcriptomics, proteomics, metabolomics) integrated with precision medicine principles and artificial intelligence (148). These approaches facilitate comprehensive investigation of molecular changes during host-parasite interactions (149), offering precise and accurate insights into host-microbe interplay. However, multi-omics technologies currently face challenges in managing large-scale population data and limitations arising from our incomplete understanding of gut microbiota and intestinal immunity—particularly in the context of personalized precision medicine. Moreover, leveraging genetically modified TSO and host models, gene editing technologies are critical for dissecting key interaction molecules and signaling pathways. These technologies may even enable the engineering of microbes to exert beneficial effects under hostile conditions. Gene editing has already been applied to single-cell organisms, particularly in probiotics research. For example, suicide plasmids are designed to have limited or no ability to replicate in the recipient bacteria, ensuring they do not persist after facilitating genetic modification and thus preventing unintended effects. This approach represents a novel and feasible concept for enhancing the biosafety of live organism therapies (150). Notably, genetically modified *Escherichia coli Nissle 1917* has demonstrated significant beneficial effects in animal models of IBD (151). Demonstrating superior therapeutic efficacy and safety profiles of this modified strain over its wild-type counterpart in human trials would mark a breakthrough in microbial therapeutics, underscoring the potential of rationally designed microorganisms to achieve optimized clinical outcomes. In helminths, beyond basic research, current practical applications of gene editing technology primarily focus on attenuating virulence in human-infecting parasites to inform infection control strategies, rather than engineering these parasites to develop therapeutic benefits (152, 153). Although gene-editing technology for helminths is currently in its early stages, this technology has undoubtedly opened the door to harnessing living organisms for disease treatment. Insights from bacterial engineering can guide the therapeutic optimization of TSO in IBD management. Ultimately, the domestication of TSO to consistently deliver therapeutic benefits represents the field’s ultimate goal.

In clinical trials, the rigorous integration of emerging foundational insights is imperative. At this stage, beyond standardizing trial design elements (e.g., sample size, auditing criteria, baseline characteristics), identifying responsive subpopulations is urgent. Stratification factors may include IBD etiology, disease severity, lesion location, and genetic predispositions. Additionally, confounding variables (e.g., concomitant medications, diet, socioeconomic factors, and psychological states) must be systematically controlled. Precision medicine principles should be systematically applied to guide the optimization of trial design. Preliminary ideas have been put into practice. In subgroup analysis from a clinical trial in 2017, it aims to determine whether clinical outcomes of TSO treatment differ between patients harboring six functional Nucleotide-binding oligomerization domain 2(NOD2) variants and wild-type counterparts, via retrospective genotyping of 207 CD patients. The result was negative.No consistent significant association was observed between the 6 NOD2 variants and clinical outcomes of TSO treatment (78). Nevertheless, this research has still taken the first step toward precision therapy of TSO for IBD.

Findings from multi - omics approaches (e.g., host-microbe interaction networks, epigenetic regulatory signatures) require prospective validation in interventional cohorts, while avoiding enrollment of heterogeneous IBD populations on large scales in single trials. Cohort studies must implement endophenotype-driven stratification to ensure cohort diversity aligns with the study objectives, accompanied by comprehensive characterization of clinical and demographic profiles (154, 155). Identifying which patient populations would benefit from a specific type of therapy would make treatment decisions more objective compared to current practices (156). This would enable clinical trials to enroll more targeted participants, thereby increasing the likelihood of successful TSO treatment. Following confirmation of safety and efficacy, genetically engineered TSO may be cautiously introduced into human trials at an appropriate stage. TSO-derived products (e.g., helminth-secreted molecules) designed to minimize confounding factors can serve as supplements to clinical trials, though most remain in preclinical stages with limited human trials (157). Additionally, TSO safety must be rigorously evaluated, including short-term risks (e.g., infection, disease exacerbation) and long-term sequelae (e.g., disease progression, carcinogenesis). Overall, the paradigm shift toward precision medicine necessitates fundamental re-engineering of clinical trial frameworks for TSO therapy in IBD. Contemporary trials should transcend conventional symptom-driven endpoints through the integration of multilayered biomarkers.

## 6 Conclusion

TSO may exert potential effects on IBD via multiple mechanisms, despite the presence of numerous knowledge gaps in this field. Despite the failure of current clinical trials to demonstrate TSO’s efficacy in IBD, its putative anti-inflammatory potential justifies continued investigation. Future trials should prioritize methodological rigor, multifactorial analysis, and target population screening. Emphasizing the role of precision medicine in TSO-based IBD research, particularly through biomarker-driven patient stratification, is likely to yield more targeted therapeutic outcomes and enhance the efficacy of clinical trials. Genomic engineering technologies possess dual potential: not only to advance the mechanistic understanding of disease pathways but also to enable the targeted domestication of TSO via precise genetic modifications. These modifications may ensure stable anti-inflammatory efficacy and predictable safety profiles in IBD immunotherapy. In addition, safety monitoring— especially in immunocompromised patients— remains paramount. These efforts aim to advance TSO from a “potential anti-inflammatory agent” to a complementary or even dominant strategy within IBD’s multidimensional treatment framework, particularly for patients refractory to conventional therapies. Once proven effective and safe over the long term, TSO could emerge as a transformative alternative to traditional drugs. Breakthroughs in this field will not only deepen our understanding of IBD pathogenesis but also propel microbiome-based interventions from empirical applications toward the precision medicine era.
